# The Relationship Between Intolerance of Uncertainty and Alcohol Use in First Responders: A Cross-Sectional Study of the Direct, Mediating and Moderating Role of Generalized Resistance Resources

**DOI:** 10.3390/ijerph22030383

**Published:** 2025-03-06

**Authors:** Tyrone B. Pretorius, Anita Padmanabhanunni

**Affiliations:** Department of Psychology, University of the Western Cape, Cape Town 7530, South Africa; apadmana@uwc.ac.za

**Keywords:** intolerance of uncertainty, alcohol use, resilience, self-compassion, self-efficacy, hardiness, moderating, mediating

## Abstract

Intolerance of uncertainty (IU) refers to the disproportionate propensity to react negatively to uncertain events, and it has been associated with negative psychological outcomes such as depression and anxiety. The current study examined the role of resilience, hardiness, self-efficacy and self-compassion in the relationship between IU and alcohol use in a sample of South African first responders. These variables are examples of what is referred to as generalized resistance resources, which enable individuals to deal with the challenges of life. A sample of 429 first responders completed the Intolerance of Uncertainty Scale-12, the Connor–Davidson Resilience Scale-10, the Self-Compassion Scale-Short Form, the General Self-Efficacy Scale, the Short Hardiness Scale and the Alcohol Use Disorders Identification Test. The results of PROCESS analyses found direct and mediating effects for resilience and self-efficacy, no effects for hardiness and moderating effects for self-compassion. In this regard, a graph of the interaction between intolerance of uncertainty and self-compassion showed that at low levels of self-compassion, the relationship between intolerance of uncertainty and alcohol use was strong and significant, while at high levels of self-compassion the relationship was non-significant. In addition, the Johnson–Neyman plot showed that the exact value of self-compassion at which point the relationship between intolerance and uncertainty ceased to be significant was 36.37. These obtained results confirm the important role of generalized resistance resources in wellbeing and provide a basis for tailored interventions.

## 1. Introduction

Intolerance of uncertainty (IU) refers to the dispositional inability to tolerate the distress arising from a perceived lack of salient or sufficient information [1]. IU is a multi-dimensional construct and comprises prospective IU and inhibitory IU. While prospective IU refers to cognitive appraisals of threat pertaining to future uncertainty, inhibitory IU entails behavioral avoidance or inhibition and difficulties in functioning when confronted with uncertainty [1]. Individuals high in IU appraise the possibility of a negative event occurring as threatening, irrespective of the likelihood of its occurrence, and experience heightened physiological arousal. This results in attempts to avoid ambiguous situations, which over time contribute to elevated levels of anxiety and impaired functioning [2,3]. IU has been implicated in a range of emotional disorders including depression, generalized anxiety disorder, post-traumatic stress disorder (PTSD), alcohol and substance use disorders, and obsessive–compulsive disorder [4,5,6,7,8]. Research undertaken during the COVID-19 pandemic highlighted that IU mediates the relationship between pandemic-related anxiety and various psychological outcomes [9,10]. For example, Karataş and Tagay found that individuals with higher IU levels experienced lower resilience and higher anxiety, which could lead to increased alcohol use as a maladaptive coping mechanism [11]. Similarly, studies have indicated that fear related to COVID-19 significantly correlates with IU and that this relationship is mediated by anxiety and depression [12,13]. The construct of IU has been investigated among diverse population groups including college students [14], adults exposed to trauma [15], healthcare workers [16], refugees [17] and war veterans [18]. The current study focused on South African first responders, specifically paramedics and police officers.

First responders are routinely exposed to life-threatening and potentially traumatic events in the course of their work. First responder groups include firefighters, paramedics, law enforcement officers and medical personnel at emergency departments and are responsible for providing multiple services during critical incidents [19,20]. In South Africa, high rates of violent crime and frequently occurring critical events such as motor vehicle accidents and fire-related incidents significantly increase the demand for first responders [21]. Existing research suggests that first responders in the country experience elevated levels of psychological distress as a consequence of duty-related exposure to potentially traumatic events [22,23]. For example, Reddy and colleagues highlight that emergency care practitioners frequently encounter traumatic situations, such as severe accidents, which can lead to psychological distress [24]. Zaida and colleagues emphasized the heavy trauma caseload faced by emergency service personnel, noting that the majority of reported injuries were intentional (e.g., gunshot and stab wounds). Ntatamala and Adams [25] noted high levels of trauma exposure among first responders and reported that 30% of their sample met the criteria for PTSD, a rate significantly higher than the 2.3% observed in the general population. South Africa also has one of the highest per capita alcohol consumption rates in Africa, which is linked to increased interpersonal violence, further exacerbating the challenges faced by first responders [26].

Existing international research has confirmed that first responders are at increased risk of developing adverse mental health outcomes as a consequence of duty-related exposure to potentially traumatic events [27,28,29]. This includes suicidal ideation, depression, generalized anxiety disorder, PTSD and heightened anger. First responders have also been identified as being susceptible to harmful or hazardous alcohol use [30]. A recent meta-analysis reported that 26% of first responders reported problematic alcohol use, a rate significantly higher than healthcare workers [31]. Kaufman and colleagues found that 56.25% of their sample of first responders met the criteria for hazardous alcohol use and highlighted its association with PTSD [20]. Additionally, Bonumwezi and colleagues found that work-related trauma exposure and the severity of PTSD symptoms were significant predictors of alcohol and drug use among first responders [32]. The self-medication hypothesis offers a framework for understanding this heightened risk and suggests that individuals may use alcohol to cope with the psychological distress and emotional dysregulation stemming from their occupational experiences [33]. Within trauma-exposed populations, IU has been identified as a factor perpetuating alcohol use disorders [3]. Individuals high in IU may experience heightened negative emotional reactions, which may in part be a consequence of elevated levels of uncertainty and hypervigilance regarding potential exposure to reminders of traumatic events. These individuals may in turn be motivated to engage in alcohol consumption as a means of reducing physiological hyperarousal and negative affect [34]. This is particularly relevant for first responders, who may face unique stressors that heighten their IU and, consequently, their reliance on alcohol for coping [34]. First responders also experience significant barriers to accessing mental healthcare, and this may further compound psychological distress. This includes perceived stigma from their peers and the community and limited access to psychological services tailored to their needs. The stigma is often compounded by the culture that emphasizes resilience and adherence to heteronormative standards of masculinity, which positions seeking help as a form of weakness. First responders often internalize these beliefs, which leads to increased shame and social isolation, further aggravating mental health challenges such as depression, anxiety and PTSD [35,36].

Despite the increased susceptibility to adverse mental health outcomes among trauma-exposed populations, existing research has highlighted the role of protective factors in promoting wellbeing [37,38]. These factors can be conceptualized as generalized resistance resources (GRRs), a central component of Antonovsky’s salutogenic model of health [39]. This model posits that health exists on a continuum from illness to wellness and that individuals possess various resources that help them navigate this continuum. GRRs are defined as any characteristics (e.g., psychological, environmental or cultural) that enable coping and adaptation and promotes wellbeing [39]. These resistance resources are known to play a crucial role in buffering the psychological impact of stressful events [40]. For instance, studies indicate that individuals with higher levels of GRRs are better equipped to handle stress and are less likely to experience mental health issues such as anxiety and depression [41,42,43]. The Job Demands–Resources model has gained significant traction in understanding the dynamics of occupational stress and wellbeing [44]. The model posits that job demands—aspects of work that require sustained physical, cognitive and emotional effort—and job resources interact to influence employee wellbeing. Job resources refer to aspects of the work environment that assist in achieving work goals, mitigating stress and promoting wellbeing (e.g., collaborative decision-making, supportive leadership and access to mental health resources) [45,46]. Within this model, GRRS can be conceptualized as factors that help individuals manage job demands and alleviate the impact of stressors.

The current study focused on the key protective factors of resilience, hardiness, self-efficacy and self-compassion. Resilience refers to the ability to adapt effectively in the context of adversity and encompasses a range of attributes, behaviors and cognitions [47]. Hardiness, self-efficacy and self-compassion represent factors that contribute to resilience. Hardiness reflects a personality trait characterized by three components, namely commitment, control and challenge [48]. Commitment reflects a sense of purpose and engagement with life, while control refers to the individual’s belief that they can influence the course of events and take active steps to address difficulties. Challenge refers to the tendency to appraise stressors as opportunities for growth rather than threats [48]. Self-efficacy refers to an individual’s belief in their ability to succeed in specific situations or accomplish a task [49] while self-compassion involves treating oneself with kindness and understanding during difficult times, rather than being overly critical or judgmental [50]. This study examined the role of resilience, hardiness, self-efficacy and self-compassion in the relationship between IU and alcohol use in a sample of first responders.

## 2. Materials and Method

### 2.1. Participants and Procedure

The participants in this study were South African first responders (*n* = 429) from the Western Cape Province of the country. The sample comprised police officers (*n* = 309) and paramedics (*n* = 120). We employed a dual recruitment strategy, namely online via Facebook groups for first responders and in-person at government hospitals and police stations. This approach ensured that participation was not limited to individuals with social media access or those who were more likely to respond to online surveys. Institutional approvals from relevant authorities facilitated in-person engagement, potentially assisting with sample diversity. We developed the online questionnaire using Google Forms and received permission from administrators of Facebook groups dedicated to first responders to post an invitation to participate and link to the questionnaire on their page. In addition to institutional ethics, we also received approval from the South African Police Service (Reference number: 3/34/2) and the Western Cape Government (Reference: WC_202307_041) to visit government hospitals and police stations to recruit first responders. The majority of the sample consisted of men (55%), were married (51.5%) and resided in an urban setting (92.3%). The mean age of the sample was 39 years (SD = 9.93) and the average length of service as a first responder was 13.23 years (SD = 9.65).

### 2.2. Measures

Participants completed a brief demographic questionnaire as well as the following instruments: the Intolerance of Uncertainty Scale-12 (IUS-12) [1], the Connor–Davidson Resilience Scale-10 (CDRISC-10) [51], the Self-Compassion Scale-Short Form (SCS-SF) [52], the General Self-Efficacy Scale (GSE) [49], the Short Hardiness Scale (SHS) [48], and the Alcohol Use Disorders Identification Test (AUDIT) [53].

The IUS-12 is, as the name indicates, a 12-item measure of intolerance of uncertainty. It is scored on a five-point scale ranging from “not all characteristic” (1) to “entirely characteristic” (5). An example of an item of the IUS-12 is “uncertainty keeps me from living a full life”. High scores on the IUS-12 indicate high levels of intolerance of uncertainty. In the original study reporting on the development of the IUS-12, Carleton and colleagues [1] reported an estimate of internal consistency of 0.91. The relationship between the IUS-12 and measures of worry, depression and anxiety provided evidence for validity. Two studies used the IUS-12 with South African entrepreneurs, and both reported satisfactory internal consistency reliability, with an alpha coefficient of 0.88 [54,55].

The CDRISC-10 is a 10-item measure of self-appraised resilience. Respondents provide their rating on a five-point scale ranging from “not true at all” (0) to “true nearly all of the time” (4). An example of a CDRISC-10 item is “I try to see the humorous side of problems”. Scores on the CDRISC-10 range from 0 to 50, with higher scores indicating higher levels of resilience. The original study reported a reliability coefficient of 0.85. The moderating role of resilience in the association between childhood maltreatment and adult psychiatric symptoms provided evidence of validity [51]. In South Africa, Pretorius and Padmanabhanunni reported satisfactory reliability (α = 0.95) for the CDRISC-10 and found that it was a valid, unidimensional measure of resilience [56].

The SCS-SF is a 12-item measure of self-compassion which can be described as compassion for oneself in the face of adversity or suffering. The SCS-SF is scored on a four-point scale ranging from “almost never” (1) to “almost always” (4). An example of an item of the SCS-SF is “when I’m going through a very hard time, I give myself the caring and tenderness I need”. Raes and colleagues [52], in the development study, reported reliability coefficients greater than 0.86 for three different samples and also confirmed the factorial validity of the SCS-SF. In South Africa, the SCS-SF has been used with students [57] as well as general employees [58], and alpha coefficients of 0.79 and 0.85 were reported for those two samples, respectively.

The GSE is a 10-item measure of perceived self-efficacy which can be described as people’s belief in their capacities to undertake and succeed in a particular task. The GSE is scored on a four-point scale with scale anchors “not at all true” (1) to “exactly true” (4). An example of an item of the GSE is “it is easy for me to stick to my aims and accomplish my goals”. Higher scores on the GSE reflect higher levels of self-efficacy. Schwarzer and Jerusalem reported reliability coefficients ranging from 0.76 to 0.90 across samples from 23 nations [49]. They also confirmed that the GSE is unidimensional. The GSE has also been used in South Africa, and one study reported a reliability coefficient of 0.66 [59].

The AUDIT is a 10-item screening instrument for harmful alcohol use and alcohol dependence. It is scored on a five-point scale with scores ranging between 0 and 40, with higher scores reflecting more harmful use of alcohol. An example of an item of AUDIT is “during the past year, how often have you found that you were not able to stop drinking once you have started?” The WHO collaborative project that developed the AUDIT reported reliability coefficients ranging between 0.80 and 0.98 across six different countries [53]. The AUDIT has been used in South Africa, and one study reported a reliability coefficient of 0.83 in a sample of university students [60].

### 2.3. Ethics

This study was conducted in accordance with the guidelines of the Declaration of Helsinki, and this study received ethical clearance from the Humanities and Social Sciences Ethics Committee of the University of the Western Cape (reference number: HS23/2/4, 23 May 2023). Participation was voluntary and anonymous, and no incentives were offered for participation. Informed consent was provided on the landing page of the electronic link.

### 2.4. Data Analyses

The data set contained no missing values as the participants could only proceed to the next page of the electronic survey if all items on the current page were responded to. We utilized IBM SPSS for Windows, version 29 (IBM Corp., Armonk, NY, USA) to obtain descriptive statistics (i.e., measures of skewness and kurtosis, means and standard deviations), estimates of internal consistency (alpha and omega) and intercorrelations between the study variables (Pearson’s r). We assessed the distribution of data using indices of skewness and kurtosis. The data are normally distributed if the skewness values range between −2 and +2 [61]. Additionally, excess kurtosis—calculated by subtracting 3 from the kurtosis value—was used to assess deviations from normality [62]. It should range between −1 and +1 [63].

The PROCESS macro [64] in SPSS was used to examine the mediating role of the generalized resistance resources (resilience, self-compassion, self-efficacy and hardiness. In this mediation analysis, IU was the independent variable and alcohol use was the dependent variable. The significance of all effects in the path analysis model was evaluated using *p*-values and 95% bootstrapped confidence intervals.

Since a variable can be both a mediator as well as a moderator [65], we also examined the moderating role of the generalized resistance resources using the PROCESS macro in SPSS. In the moderation analysis, an interaction term consisting of the independent variable and the presumed moderator (e.g., IU X resilience) is created and a significant interaction effect is regarded as indicative of moderation. To avoid issues with multicollinearity, the two variables are mean-centered prior to creating the product term. The nature of the interaction effect is examined by plotting and comparing a regression line for three different values of the moderator: 1SD below the mean, at the mean and 1SD above the mean. In addition, we used a freely available Excel spreadsheet, CAHOST [66], to implement the Johnson–Neyman technique [67]. This procedure determines the exact value of the moderator variable where the relationship between the independent and dependent variable ceases to be significant.

## 3. Results

The descriptive statistics (means, standard deviations, indices of skewness and kurtosis), reliabilities (alpha and omega) and intercorrelations between variables are reported in Table 1.

Table 1 indicates that the scores produced by all of the instruments demonstrated satisfactory and acceptable estimates of internal consistency (α and ω = 0.83–0.94). The indices of skewness ranged between −0.61 and 1.14, while the indices of kurtosis ranged between −0.31 and 0.60. These indices fell within an acceptable range (skewness: −2 and +2; kurtosis: −1 and +1), thus demonstrating that the data for all variables is approximately normally distributed.

Table 1 also shows that the generalized resistance resources were positively correlated with each other. In the case of the relationships between resilience and hardiness (*r* = 0.51, *p* < 0.001) and self-efficacy (*r* = 0.63, *p* < 0.001), as well as the relationship between hardiness and self-efficacy, the obtained relationships may be described as a large effect size (>0.50). On the other hand, for the relationships between self-compassion and resilience (*r* = 0.26, *p* < 0.001), hardiness (*r* = 0.26, *p* < 0.001) and self-efficacy (*r* = 0.32, *p* < 0.001), the obtained coefficients may be described as medium effect sizes (0.30–0.50). Alcohol use was significantly negatively related to resilience (*r* = −0.17, *p* < 0.001) and self-efficacy (*r* = −0.15, *p* = 0.002) and significantly positively related to self-compassion (*r* = 0.11, *p* = 0.02) but not associated with alcohol use (*r* = 0.09, *p* < 0.001).

The direct, mediating and moderating effects of resilience, hardiness, self-efficacy and self-compassion are reported in Table 2.

Table 2 indicates, firstly, that only resilience (95% CI [−0.35, −0.12]) and self-efficacy (95% CI [−0.44, −0.12]) had direct effects on alcohol use. The negative standardized coefficients indicate that higher levels of resilience and self-efficacy are associated with lower levels of alcohol use.

**Figure 1:**
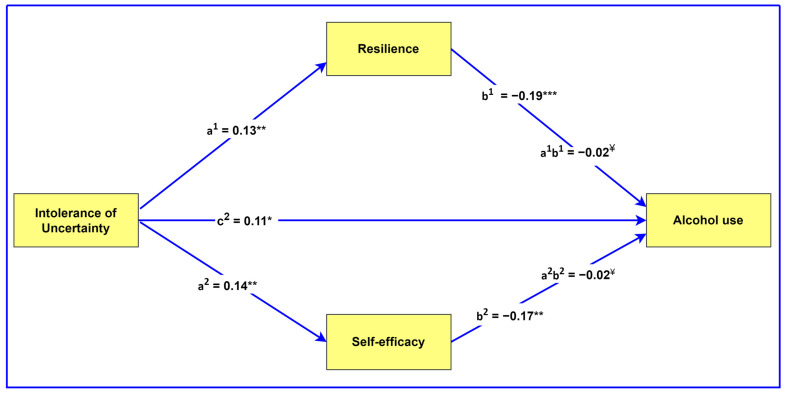
Visual representation of the mediating roles of resilience and self-efficacy. All regression coefficients are standardized. a^1^ and a^2^ = direct effects of independent variable on mediators. c^2^ = effect of independent variable on dependent variable. b^1^ and b^2^ = direct effects of mediators on dependent variable. a^1^b^1^ and a^2^b^2^ = mediating effects. * *p* < 0.05, ** *p* < 0.01, *** *p* 0.001, ^¥^ 95% confidence intervals.

Table 2, secondly, shows that resilience (95% CI [−0.05, −0.00]) and self-efficacy (95% CI [−0.05, −0.00]) mediated the relationship between IU and alcohol use. Since the association between IU and alcohol use was significant in the presence of the mediators (resilience: β = 0.11, *p* = 0.018; self-efficacy: β = 0.11, *p* = 0.200), it would indicate that resilience and self-efficacy partially mediated the relationship between IU and alcohol use. The mediation effects are visually demonstrated in Figure 1.

**Figure 2:**
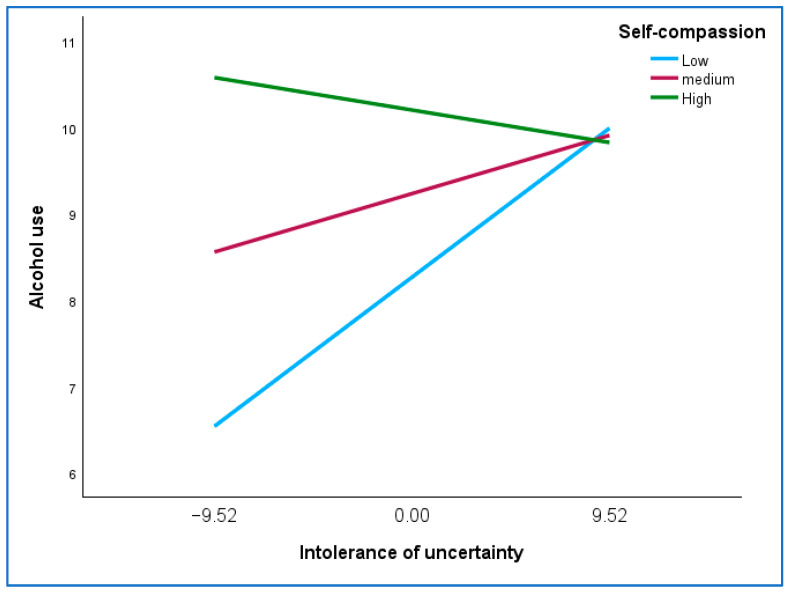
The relationship between intolerance of uncertainty and alcohol use for low, medium and high self-compassion.

Thirdly, Table 2 shows that hardiness had no direct, mediating or moderating effect, while self-compassion only had a moderating effect. The nature of the moderating role of self-compassion in the relationship between IU and alcohol use is reported in Table 3 and visually shown in Figure 2.

Table 3 shows that at a low value of self-compassion (1 SD below the mean), the association between IU and alcohol use was statistically significant (*p* = 0.012), while at higher values of self-compassion (mean and 1SD above the mean), there was no significant relationship between IU and alcohol use. This is illustrated in Figure 2, which shows the plots of the regression lines of the relationship between IU and alcohol use for the three values of self-compassion (1SD below the mean, the mean, 1SD above the mean).

**Figure 3:**
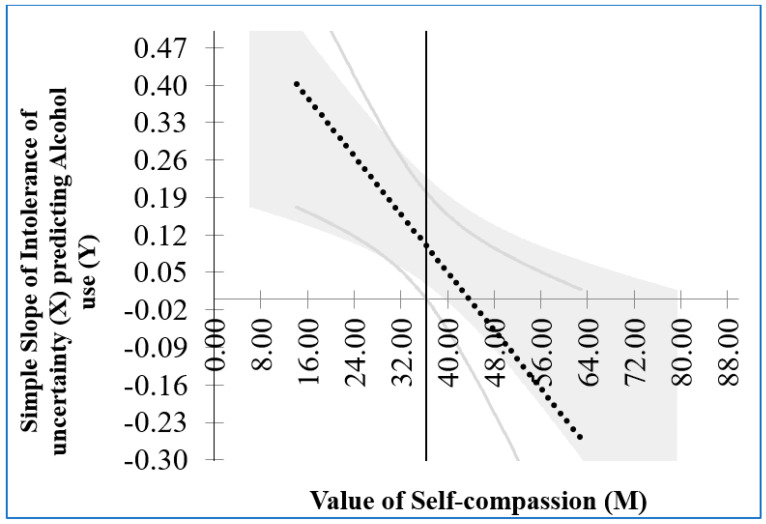
Johnson–Neyman plot identifying the self-compassion score where the relationship between IU and alcohol use is no longer significant.

Figure 2 shows an increase in the relationship between IU and alcohol use at low levels of self-compassion (positive association) and a decrease in the relationship for those high in self-compassion (negative association). At low levels of IU, the relationship between IU and alcohol use was stronger for those high in self-compassion than for those low in self-compassion. However, at high levels of IU, there is a cross-over effect where the relationship between IU and alcohol use is stronger for those low in self-compassion than for those high in self-compassion. Figure 3 shows the Johnson–Neyman plot that identifies the exact self-compassion score where the relationship between IU and alcohol use is no longer significant.

In Figure 3, the grey area represents the confidence interval for the association between IU and alcohol use. The point where the confidence interval contains zero (i.e., where the relationship is no longer significant) is indicated by the vertical line between the scores of 32 and 40 on the X-axis. The exact value indicated by the Johnson–Neyman graph is 36.37.

## 4. Discussion

First responders frequently encounter potentially traumatic events at substantially higher rates than the general population. This enhances their vulnerability to negative mental health outcomes, notably PTSD, depression and alcohol use disorder [68,69]. The current study examined the role of resilience, hardiness, self-efficacy and self-compassion in the relationship between IU and alcohol use in a sample of South African police officers and paramedics. There were several relevant findings. First, resilience and self-efficacy emerged as protective factors, in that higher levels of these GRRs were associated with lower levels of alcohol use. This finding aligns with the existing literature. For instance, a South African study reported that lower levels of resilience were associated with a history of alcohol use among ambulance personnel [70]. Pachi and colleagues found that psychological resilience mediated the relationship between anger and aggression and alcohol abuse among health professionals [71]. Furthermore, Safiye and colleagues demonstrated that resilience significantly contributes to mental health outcomes among healthcare workers, with higher levels of resilience associated with lower levels of depression, anxiety and stress [72]. This corresponds with the established literature indicating that resilient individuals are better equipped to manage stress and are less likely to engage in harmful behaviors. Research on self-efficacy has underscored its role as a protective factor. Yin and colleagues, for example, found that self-efficacy had a mediating role in the association between social support and resilience in patients undergoing lung cancer treatment. Their findings suggest that those with higher self-efficacy are better equipped to manage treatment-related stressors [73]. In a study on school teachers during the COVID-19 pandemic, Li reported that self-efficacy was negatively associated with burnout. This study highlighted that belief in one’s ability to manage specific challenges is a salient resource in promoting mental health [74].

Second, resilience and self-efficacy partially mediated the relationship between IU and alcohol use, indicating that these GRRs act as mechanisms through which IU influences alcohol consumption. This aligns with the extant literature base on the mediating role of protective factors. Wang and colleagues, for instance, found that coping style mediated the relationship between IU and anxiety among college students [75]. Similarly, Wahesh and Ondrejack reported that coping motives mediated the relationship between inhibitory IU and alcohol-related problems among Turkish workers [76]. Yang and colleagues explored the relationship between self-control, resilience and self-efficacy among individuals with substance use disorders and reported that resilience mediated the relationship between self-control and self-efficacy [77]. This suggests that fostering resilience can enhance self-efficacy by potentially creating a positive feedback loop that supports recovery and personal growth.

Third, self-compassion moderated the relationship between IU and alcohol use. There was an increase in the relationship between IU and alcohol use at low levels of self-compassion (positive association) and a decrease in the relationship for those high in self-compassion (negative association). This finding is supported by studies that have highlighted the role of self-compassion in fostering emotional regulation and promoting psychological wellbeing. A study on college teachers found that self-compassion significantly mediated the relationship between psychological resilience and mental health [78]. The findings suggest that self-compassion helps to reduce self-criticism and ruminative thought processes and facilitates cognitive re-appraisal of maladaptive cognitions, which are associated with anxiety, alcohol use and depression. By fostering self-kindness and a balanced emotional outlook, self-compassion helps buffer the negative impact of stressful life events, thereby promoting mental health [78]. Similarly, Zhang and Shen explored the role of self-compassion among Chinese college students during the COVID-19 pandemic, finding that self-compassion mediated the relationship between dispositional mindfulness and mental health [79].

Interestingly, the current study found that at low levels of IU, the relationship between IU and alcohol use was stronger for those high in self-compassion compared to those low in self-compassion. However, at high levels of IU, there is a cross-over effect where the relationship between IU and alcohol use is stronger for those low in self-compassion than for those high in self-compassion. This finding suggests that self-compassion operates differently depending on the degree of IU. At low levels of IU, individuals with higher self-compassion may be more attuned to their emotional experiences and the challenges posed by uncertainty. This heightened awareness could, in some cases, lead to an increased likelihood of using alcohol as a temporary coping mechanism for emotional distress, especially if other coping strategies are unavailable. In this context, self-compassion might amplify awareness of the stressor and its potential implications, inadvertently reinforcing the use of alcohol to manage discomfort. In contrast, at high levels of IU, self-compassion seems to exert a protective effect. Individuals with high self-compassion are likely better equipped to regulate their emotions and respond to intense uncertainty in adaptive ways. They may rely on self-kindness, mindfulness, and a balanced perspective, which reduce the likelihood of turning to alcohol as a coping strategy. Conversely, those with low self-compassion may lack these regulatory resources, leaving them more vulnerable to the distress caused by high IU and more likely to engage in maladaptive coping behaviors, such as alcohol use. The identified threshold of a self-compassion score of 36.37 highlights the point at which self-compassion effectively neutralizes the impact of IU on alcohol use. This suggests that interventions aimed at increasing self-compassion could be particularly beneficial for individuals experiencing high levels of IU, as even moderate improvements in self-compassion may significantly mitigate the risk of alcohol misuse.

The findings of the current study have important implications for designing interventions aimed at reducing alcohol use, particularly among populations that are routinely exposed to potentially traumatic events in the course of their work. GRRs are modifiable factors that can be enhanced through appropriate intervention. Interventions that aim to cultivate self-compassion, such as compassion-focused therapy, could be particularly effective among first responders [80]. These approaches encourage individuals to adopt a kinder, less critical stance toward themselves; promote emotional regulation; and foster resilience to distress caused by uncertainty [81]. Given the identified threshold of self-compassion at which the relationship between IU and alcohol use becomes non-significant, intervention programmes should aim to help participants achieve and sustain self-compassion levels above this critical point. Tailored strategies, such as guided mindfulness exercises, psychoeducation on the benefits of self-compassion and cognitive restructuring to challenge self-critical thought patterns, could be integrated into these programmes [81]. The findings also highlight the need to address IU as a key driver of alcohol use among first responders. Cognitive-behavioral interventions that focus on increasing tolerance for uncertainty, such as exposure-based approaches or Acceptance and Commitment Therapy (ACT), may help individuals develop adaptive responses to uncertainty and reduce reliance on maladaptive coping strategies such as substance and alcohol use. Cognitive-behavioral strategies can also enhance self-efficacy by enabling individuals to reframe negative self-beliefs and develop more positive, adaptive thought patterns. Evidence-based interventions that integrate these components could provide first responders with the tools they need to manage occupational stress more effectively, ultimately supporting their mental health and their ability to perform their critical roles in society. For clinicians, integrating exposure-based interventions and ACT could provide more effective ways to assist first responders in managing distress without resorting to alcohol use. Furthermore, workplace-based mental health programmes tailored for first responders should incorporate psychoeducation on resilience-building and self-efficacy enhancement, as these factors may buffer against the negative psychological impacts of occupational trauma. Given the identification of a self-compassion threshold (36.37), interventions aimed at helping first responders reach and maintain this level of self-compassion could be particularly beneficial.

This study had several limitations. A cross-sectional design was used, which limits the ability to infer causality. While associations between IU, GRRs and alcohol use were observed, the temporal relationships between these variables remain unclear. Longitudinal studies are needed to better understand how these relationships develop over time. Data collection relied exclusively on self-report questionnaires, which are subject to biases such as social desirability, recall errors and self-perception inaccuracies. This limitation is particularly relevant in studies involving first responders, where stigma surrounding mental health and substance use may influence participants’ responses. While voluntary participation carries the potential for self-selection bias, the combined recruitment strategy potentially increased the likelihood of obtaining a broader representation of first responders in the Western Cape. The sample consisted of first responders primarily from a specific geographical region, potentially limiting the generalizability of the findings to other populations or contexts. It is possible that confounding variables may have influenced our findings (e.g., work-related stressors, organizational support and personality-related dimensions). Future studies should consider these potential confounders to enhance the robustness of findings. Although this study identified a specific self-compassion threshold (36.37) at which the relationship between IU and alcohol use became non-significant, this finding may vary depending on the sample and measurement tools used. Replication studies are needed to confirm and refine this threshold in diverse populations.

## 5. Conclusions

This study highlights the critical role of GRRs in the relationship between IU and alcohol use among South African first responders. The results provide a strong foundation for developing tailored interventions aimed at enhancing GRRs, such as resilience training, self-compassion practices and cognitive-behavioral strategies to increase IU tolerance. By addressing these modifiable factors, interventions can help mitigate alcohol use and promote overall wellbeing among first responders.

## Figures and Tables

**Table 1:** Descriptive statistics, reliabilities and intercorrelations between study variables.

Scale/Variable	1	2	3	4	5	6
1. IU	—					
2. Resilience	0.13 **	—				
3. Hardiness	0.27 ***	0.51 ***	—			
4. Self-efficacy	0.14 **	0.63 ***	0.63 ***	—		
5. Self-compassion	0.35 ***	0.26 ***	0.40 ***	0.32 ***	—	
6. Alcohol use	0.09	−0.17 ***	−0.02	−0.15 **	0.11 *	—
Mean	35.72	26.48	26.34	30.45	38.49	8.87
SD	9.51	8.02	8.37	5.93	8.14	10.01
Skewness	0.17	−0.40	0.07	−0.61	0.01	1.14
Kurtosis	−0.31	−0.19	−0.07	0.36	0.11	0.60
Alpha	0.89	0.92	0.88	0.91	0.84	0.94
Omega	0.89	0.92	0.88	0.91	0.83	0.94

Note. IU = Intolerance of uncertainty. * *p* < 0.05, ** *p* < 0.01, *** *p* < 0.001.

**Table 2:** The direct, mediating and moderating effects of resilience, hardiness, self-efficacy and self-compassion.

Effect Type	Effect	B	SE	95% CI	β	*p*
Direct effects	resilience → alcohol use	−0.23	0.06	[−0.35, −0.12] *	−0.19	<0.001
	hardiness → alcohol use	−0.05	0.06	[−0.17, 0.07]	−0.04	0.408
	self-efficacy → alcohol use	−0.28	0.08	[−0.44, −0.12] *	−0.17	0.001
	self-compassion → alcohol use	0.11	0.06	[−0.01, 0.24]	0.09	0.072
Mediating effects	IU → resilience → alcohol use	−0.03	0.01	[−0.05, −0.00] *	−0.02	—
	IU → hardiness → alcohol use	−0.01	0.02	[−0.04. 0.02]	−0.01	—
	IU → self-efficacy → alcohol use	−0.02	0.01	[−0.05, −0.00] *	−0.02	—
	IU → self-compassion → alcohol use	0.03	0.02	[−0.04, 0.08]	0.03	—
Moderating effects	IU X resilience → alcohol use	0.00	0.01	[−0.01, 0.02]	0.03	>0.05
	IU X hardiness → alcohol use	−0.01	0.01	[−0.02, 0.01]	−0.04	0.417
	IU X self-efficacy → alcohol use	0.00	0.01	[−0.01, 0.02]	0.01	0.881
	IU X self-compassion → alcohol use	−0.01	0.01	[−0.02, −0.00] *	−0.11	0.013

Note. IU = intolerance of uncertainty. * Confidence intervals that do not include zero and thus reflect a statistically significant effect.

**Table 3:** The association between intolerance of uncertainty and alcohol use at different values of self-compassion.

Self-Compassion	Effect	SE	95% CI	*p*
1 standard deviation below the mean	0.18	0.07	[0.04, 0.32]	0.012
Mean	0.07	0.05	[−0.04, 0.18]	0.189
1 standard deviation above the mean	−0.04	0.07	[−0.17, 0.09]	0.555

## Data Availability

The original data presented in this study are openly available in UWCScholar at https://uwcscholar.uwc.ac.za/items/2a66a4d5-b281-40c0-b137-3d5952953ed5 (accessed on 19 January 2025).

## References

[1] Carleton R.N., Norton M.A.P.J., Asmundson G.J.G. (2007). Fearing the unknown: A short version of the Intolerance of Uncertainty Scale. J. Anxiety Disord..

[2] Oglesby M.E., Gibby B.A., Mathes B.M., Short N.A., Schmidt N.B. (2017). Intolerance of Uncertainty and Post-traumatic Stress Symptoms: An Investigation within a Treatment Seeking Trauma-Exposed Sample. Compr. Psychiatry.

[3] Paltell K.C., Edalatian Zakeri S., Gorka S.M., Berenz E.C. (2022). PTSD Symptoms, Intolerance of Uncertainty, and Alcohol-Related Outcomes Among Trauma-Exposed College Students. Cogn. Ther. Res..

[4] Rettie H., Daniels J. (2021). Coping and Tolerance of Uncertainty: Predictors and Mediators of Mental Health During the COVID-19 Pandemic. Am. Psychol..

[5] Wheaton M.G., Messner G.R., Marks J.B. (2021). Intolerance of uncertainty as a factor linking obsessive-compulsive symptoms, health anxiety and concerns about the spread of the novel coronavirus (COVID-19) in the United States. J. Obs.-Compuls. Relat. Disord..

[6] Pinciotti C.M., Riemann B.C., Abramowitz J.S. (2021). Intolerance of uncertainty and obsessive-compulsive disorder dimensions. J. Anxiety Disord..

[7] Venanzi L., Dickey L., Green H., Pegg S., Benningfield M.M., Bettis A.H., Blackford J.U., Kujawa A. (2022). Longitudinal predictors of depression, anxiety, and alcohol use following COVID-19-related stress. Stress Health.

[8] Bottesi G., Ghisi M., Caggiu I., Lauriola M. (2021). How is intolerance of uncertainty related to negative affect in individuals with substance use disorders? The role of the inability to control behaviors when experiencing emotional distress. Addict. Behav..

[9] Andrews J.L., Li M., Minihan S., Songco A., Fox E., Ladouceur C.D., Mewton L., Moulds M., Pfeifer J.H., Van Harmelen A.-L. (2023). The effect of intolerance of uncertainty on anxiety and depression, and their symptom networks, during the COVID-19 pandemic. BMC Psychiatry.

[10] Chung S., Lee T., Hong Y., Ahmed O., Silva W.A.D., Gouin J.-P. (2022). Viral Anxiety Mediates the Influence of Intolerance of Uncertainty on Adherence to Physical Distancing Among Healthcare Workers in COVID-19 Pandemic. Front. Psychiatry.

[11] Karataş Z., Tagay Ö. (2021). The relationships between resilience of the adults affected by the covid pandemic in Turkey and COVID-19 fear, meaning in life, life satisfaction, intolerance of uncertainty and hope. Pers. Individ. Differ..

[12] Kardas F. (2021). The Fear of COVID-19 Raises the Level of Depression, Anxiety and Stress through the Mediating Role of Intolerance of Uncertainty. Stud. Psychol..

[13] Voitsidis P., Nikopoulou V.A., Holeva V., Parlapani E., Sereslis K., Tsipropoulou V., Karamouzi P., Giazkoulidou A., Tsopaneli N., Diakogiannis I. (2021). The mediating role of fear of COVID-19 in the relationship between intolerance of uncertainty and depression. Psychol. Psychother..

[14] Lauriola M., Carleton R.N., Tempesta D., Calanna P., Socci V., Mosca O., Salfi F., De Gennaro L., Ferrara M. (2019). A Correlational Analysis of the Relationships among Intolerance of Uncertainty, Anxiety Sensitivity, Subjective Sleep Quality, and Insomnia Symptoms. Int. J. Environ. Res. Public Health.

[15] Arditte Hall K.A., Arditte S.J. (2024). Threat-Related Interpretation Biases and Intolerance of Uncertainty in Individuals Exposed to Trauma. Cogn. Ther. Res..

[16] Yıldırım M., Çağış Z.G., Gómez-Salgado J. (2024). Intolerance of Uncertainty, Job Satisfaction and Work Performance in Turkish Healthcare Professionals: Mediating Role of Psychological Capital. Int. J. Public Health.

[17] Nickerson A., Hoffman J., Keegan D., Kashyap S., Argadianti R., Tricesaria D., Pestalozzi Z., Nandyatama R., Khakbaz M., Nilasari N. (2023). Intolerance of uncertainty, posttraumatic stress, depression, and fears for the future among displaced refugees. J. Anxiety Disord..

[18] Clauss K., Houtsma C., Shapiro M.O., McDermott M.J., Macia K.S., Franklin C.L., Raines A.M., Ferreira R.J. (2024). Anxiety Sensitivity and Intolerance of Uncertainty Among Veterans with Subthreshold Versus Threshold PTSD. Traumatology.

[19] Benincasa V., Passannante M., Pierrini F., Carpinelli L., Moccia G., Marinaci T., Capunzo M., Pironti C., Genovese A., Savarese G. (2022). Burnout and Psychological Vulnerability in First Responders: Monitoring Depersonalization and Phobic Anxiety during the COVID-19 Pandemic. Int. J. Environ. Res. Public Health.

[20] Kaufman C.C., Vujanovic A.A., Murphy J.G., Rosmarin D.H. (2024). The association between PTSD symptom clusters and religion/spirituality with alcohol use among first responders. J. Psychiatr. Res.

[21] Delaney P.G., Eisner Z.J., Bustos A., Hancock C.J., Thullah A.H., Jayaraman S., Raghavendran K. (2022). Cost-Effectiveness of Lay First Responders Addressing Road Traffic Injury in Sub-Saharan Africa. J. Surg. Res..

[22] O’Neil J.W., Kruger L. (2022). Mindset as a resilience resource and perceived wellness of first responders in a South African context. Jamba.

[23] Padmanabhanunni A., Pretorius T.B. (2024). Being Cynical Is Bad for Your Wellbeing: A Structural Equation Model of the Relationship Between Cynicism and Mental Health in First Responders in South Africa. Int. J. Environ. Res. Public Health.

[24] Reddy L., Moodley I., Muslim T.A. (2022). Knowledge, attitudes and practices of emergency care practitioners in the management of common dental emergencies in the eThekwini District, KwaZulu-Natal. S. Afr. Dent. J..

[25] Ntatamala I., Adams S. (2022). The correlates of post-traumatic stress disorder in ambulance personnel and barriers faced in accessing care for work-related stress. Int. J. Environ. Res. Public Health.

[26] Bartlett A., Lesch M., Golder S., McCambridge J. (2023). Alcohol policy framing in South Africa during the early stages of COVID-19: Using extraordinary times to make an argument for a new normal. BMC Public Health.

[27] Phillips W.J., Cocks B.F., Manthey C., Kendall-Tackett K., Kendall-Tackett K.A. (2023). Ambulance Ramping Predicts Poor Mental Health of Paramedics. Psychol. Trauma.

[28] Stogner J., Miller B.L., McLean K. (2020). Police Stress, Mental Health, and Resiliency during the COVID-19 Pandemic. Am. J. Crim. Justice.

[29] Syed S., Ashwick R., Schlosser M., Jones R., Rowe S., Billings J. (2020). Global prevalence and risk factors for mental health problems in police personnel: A systematic review and meta-analysis. Occup. Environ. Med..

[30] Gryshchuk L., Campbell M.A., Brunelle C., Doyle J.N., Nero J.W. (2022). Profiles of Vulnerability to Alcohol Use and Mental Health Concerns in First Responders. J. Police Crim. Psychol..

[31] Irizar P., Puddephatt J.-A., Gage S.H., Fallon V., Goodwin L. (2021). The prevalence of hazardous and harmful alcohol use across trauma-exposed occupations: A meta-analysis and meta-regression. Drug Alcohol Depend..

[32] Bonumwezi J.L., Tramutola D., Lawrence J., Kobezak H.M., Lowe S.R. (2022). Posttraumatic stress disorder symptoms, work-related trauma exposure, and substance use in first responders. Drug Alcohol Depend..

[33] Khantzian E.J. (1997). The self-medication hypothesis of substance use disorders: A reconsideration and recent applications. Harv. Rev. Psychiatry.

[34] Bilewicz M., Babińska M., Gromova A. (2024). High rates of probable PTSD among Ukrainian war refugees: The role of intolerance of uncertainty, loss of control and subsequent discrimination. Eur. J. Psychotraumatol..

[35] Arjmand H.-A., O’Donnell M.L., Putica A., Sadler N., Peck T., Nursey J., Varker T., Kearney L.K. (2024). Mental Health Treatment for First Responders: An Assessment of Mental Health Provider Needs. Psychol. Serv..

[36] Haugen P.T., McCrillis A.M., Smid G.E., Nijdam M.J. (2017). Mental health stigma and barriers to mental health care for first responders: A systematic review and meta-analysis. J. Psychiatr. Res.

[37] Kagee A., Padmabhanunni A., Coetzee B., Booysen D., Kidd M. (2024). Sense of coherence, social support, satisfaction with life, and resilience as mediators between fear of COVID-19, perceived vulnerability to disease and depression. S. Afr. J. Psychol..

[38] Danioni F., Barni D., Ferrari L., Ranieri S., Canzi E., Iafrate R., Lanz M., Regalia C., Rosnati R. (2023). The enduring role of sense of coherence in facing the pandemic. Health Promot. Int..

[39] Antonovsky A. (1996). The salutogenic model as a theory to guide health promotion. Health Promot. Int..

[40] Padmanabhanunni A., Isaacs S., Pretorius T., Faroa B. (2022). Generalized Resistance Resources in the Time of COVID-19: The Role of Sense of Coherence and Resilience in the Relationship between COVID-19 Fear and Loneliness among Schoolteachers. OBM Neurobiol..

[41] Mana A., Bauer G.F., Meier Magistretti C., Sardu C., Juvinyà-Canal D., Hardy L.J., Catz O., Tušl M., Sagy S. (2021). Order out of chaos: Sense of coherence and the mediating role of coping resources in explaining mental health during COVID-19 in 7 countries. SSM-Ment. Health.

[42] Super S., Pijpker R., Polhuis K. (2021). The relationship between individual, social and national coping resources and mental health during the COVID-19 pandemic in the Netherlands. Health Psychol. Rep..

[43] Li Z.-S., Hasson F. (2020). Resilience, stress, and psychological well-being in nursing students: A systematic review. Nurse Educ. Today.

[44] Bakker A.B., Demerouti E. (2007). The Job Demands-Resources model: State of the art. J. Manag. Psychol..

[45] Santa Maria A., Wörfel F., Wolter C., Gusy B., Rotter M., Stark S., Kleiber D., Renneberg B. (2018). The Role of Job Demands and Job Resources in the Development of Emotional Exhaustion, Depression, and Anxiety Among Police Officers. Police Q..

[46] Han J., Yin H., Wang J., Zhang J. (2020). Job demands and resources as antecedents of university teachers’ exhaustion, engagement and job satisfaction. Educ. Psychol..

[47] Connor K.M., Davidson J.R.T. (2003). Development of a new resilience scale: The Connor-Davidson Resilience Scale (CD-RISC). Depress. Anxiety.

[48] Bartone P.T. (1995). A Short Hardiness Scale.

[49] Schwarzer R., Jerusalem M., Johnston M., Wright S., Weinman J. (1995). Generalized self-efficacy scale. Measures in Health Psychology: A User’s Portfolio.

[50] Neff K.D. (2011). Self-Compassion, Self-Esteem, and Well-Being. Soc. Pers. Psychol. Compass.

[51] Campbell-Sills L., Stein M.B. (2007). Psychometric analysis and refinement of the connor-davidson resilience scale (CD-RISC): Validation of a 10-item measure of resilience. J. Trauma. Stress.

[52] Raes F., Pommier E., Neff K.D., Van Gucht D. (2011). Construction and factorial validation of a short form of the Self-Compassion Scale. Clin. Psychol. Psychother..

[53] Saunders J.B., Aasland O.G., Babor T.F., De La Fuente J.R., Grant M. (1993). Development of the alcohol use disorders identification test (AUDIT): WHO collaborative project on early detection of persons with harmful alcohol consumption-II. Addiction.

[54] Tran T. (2020). Intolerance of Uncertainty and Cultural Tightness-Looseness: Two Antecedents of Effectuation-Causation: Evidence from South Africa. Master’s Thesis.

[55] Vreugdenhil H.G. (2020). To Predict, Or to Control That Is the Question: The Influence of Intolerance of Uncertainty on Entrepreneurial Decision-Making Behaviour. Master’s Thesis.

[56] Pretorius T.B., Padmanabhanunni A. (2022). Validation of the Connor-Davidson Resilience Scale-10 in South Africa: Item Response Theory and Classical Test Theory. Psychol. Res. Behav. Manag..

[57] Walker S. (2021). Self-compassion mediates the relationship between dispositional mindfulness and athlete burnout among adolescent squash players in South Africa. S. Afr. J. Sports Med..

[58] Kotera Y., Mayer C.-H., Vanderheiden E. (2021). Cross-Cultural Comparison of Mental Health Between German and South African Employees: Shame, Self-Compassion, Work Engagement, and Work Motivation. Front. Psychol..

[59] Redelinghuys J.R. (2010). General Self–Efficacy as a Moderator Between Stress and Positive Mental Health in an African Context. Master’s Thesis.

[60] Young C., Mayson T. (2010). The Alcohol Use Disorders Identification Scale (AUDIT) normative scores for a multiracial sample of Rhodes University residence students. J. Child Adolesc. Ment. Health.

[61] Hair J.F., Black W.C., Babin B.J., Anderson R.E. (2010). Multivariate Data Analysis.

[62] Kim H.-Y. (2013). Statistical notes for clinical researchers: Assessing normal distribution (2) using skewness and kurtosis. Restor. Dent. Endod..

[63] Taylor Enterprises Excess Kurtosis. https://variation.com/wp-content/distribution_analyzer_help/hs139.htm.

[64] Hayes A.F. (2017). Introduction to Mediation, Moderation, and Conditional Process Analysis: A Regression-Based Approach.

[65] Judd C.M., Kenny D.A., McClelland G.H. (2001). Estimating and Testing Mediation and Moderation in Within-Subject Designs. Psychol. Methods.

[66] Carden S.W., Holtzman N.S., Strube M.J. (2017). CAHOST: An Excel Workbook for Facilitating the Johnson-Neyman Technique for Two-Way Interactions in Multiple Regression. Front. Psychol..

[67] Johnson P.O., Neyman J. (1936). Tests of certain linear hypotheses and their application to some educational problems. Stat. Res. Mem..

[68] Lanza A., Roysircar G., Rodgers S. (2018). First responder mental healthcare: Evidence-based prevention, postvention, and treatment. Prof. Psychol. Res. Pract..

[69] McAlearney A.S., Gaughan A.A., MacEwan S.R., Gregory M.E., Rush L.J., Volney J., Panchal A.R. (2022). Pandemic experience of first responders: Fear, frustration, and stress. Int. J. Environ. Res. Public Health.

[70] McIzana T., Adams S., Khan S., Ntatamala I. (2024). Sociodemographic and work-related factors associated with psychological resilience in South African healthcare workers: A cross-sectional study. BMC Health Serv. Res..

[71] Pachi A., Kavourgia E., Bratis D., Fytsilis K., Papageorgiou S.M., Lekka D., Sikaras C., Tselebis A. (2023). Anger and Aggression in Relation to Psychological Resilience and Alcohol Abuse among Health Professionals during the First Pandemic Wave. Healthcare.

[72] Safiye T., Gutić M., Dubljanin J., Stojanović T.M., Dubljanin D., Kovačević A., Zlatanović M., Demirović D.H., Nenezić N., Milidrag A. (2023). Mentalizing, Resilience, and Mental Health Status among Healthcare Workers during the COVID-19 Pandemic: A Cross-Sectional Study. Int. J. Environ. Res. Public Health.

[73] Yin Y., Lyu M., Chen Y., Zhang J., Li H., Li H., Xia G., Zhang J. (2022). Self-efficacy and positive coping mediate the relationship between social support and resilience in patients undergoing lung cancer treatment: A cross-sectional study. Front. Psychol..

[74] Li S. (2023). The effect of teacher self-efficacy, teacher resilience, and emotion regulation on teacher burnout: A mediation model. Front. Psychol..

[75] Wang T., Jiang L., Li T., Zhang X., Xiao S. (2023). The relationship between intolerance of uncertainty, coping style, resilience, and anxiety during the COVID-19 relapse in freshmen: A moderated mediation model. Front. Psychiatry.

[76] Wahesh E., Ondrejack L. (2024). Intolerance of uncertainty dimensions and alcohol problems: The effects of coping motives and heavy drinking. J. Addict. Offender Couns..

[77] Yang C., Zhou Y., Cao Q., Xia M., An J. (2019). The Relationship Between Self-Control and Self-Efficacy Among Patients With Substance Use Disorders: Resilience and Self-Esteem as Mediators. Front. Psychiatry.

[78] Rehman S., Addas A., Rehman E., Khan M.N. (2024). The Mediating Roles of Self-Compassion and Emotion Regulation in the Relationship Between Psychological Resilience and Mental Health Among College Teachers. Psychol. Res. Behav. Manag..

[79] Zhang D., Shen J. (2023). Dispositional mindfulness and mental health among Chinese college students during the COVID-19 lockdown: The mediating role of self-compassion and the moderating role of gender. Front. Psychol..

[80] Petrocchi N., Ottaviani C., Cheli S., Matos M., Baldi B., Basran J.K., Gilbert P., Nezu A.M. (2024). The Impact of Compassion-Focused Therapy on Positive and Negative Mental Health Outcomes: Results of a Series of Meta-Analyses. Clin. Psychol..

[81] Wakelin K.E., Perman G., Simonds L.M. (2022). Effectiveness of self-compassion-related interventions for reducing self-criticism: A systematic review and meta-analysis. Clin. Psychol. Psychother..

